# Supplementary figures and images for: Distribution-preserving data augmentation
Source: PeerJ Comput Sci. 2021 May 27;7:e571. doi: 10.7717/peerj-cs.571 (PMC8176531; doi:10.7717/peerj-cs.571)

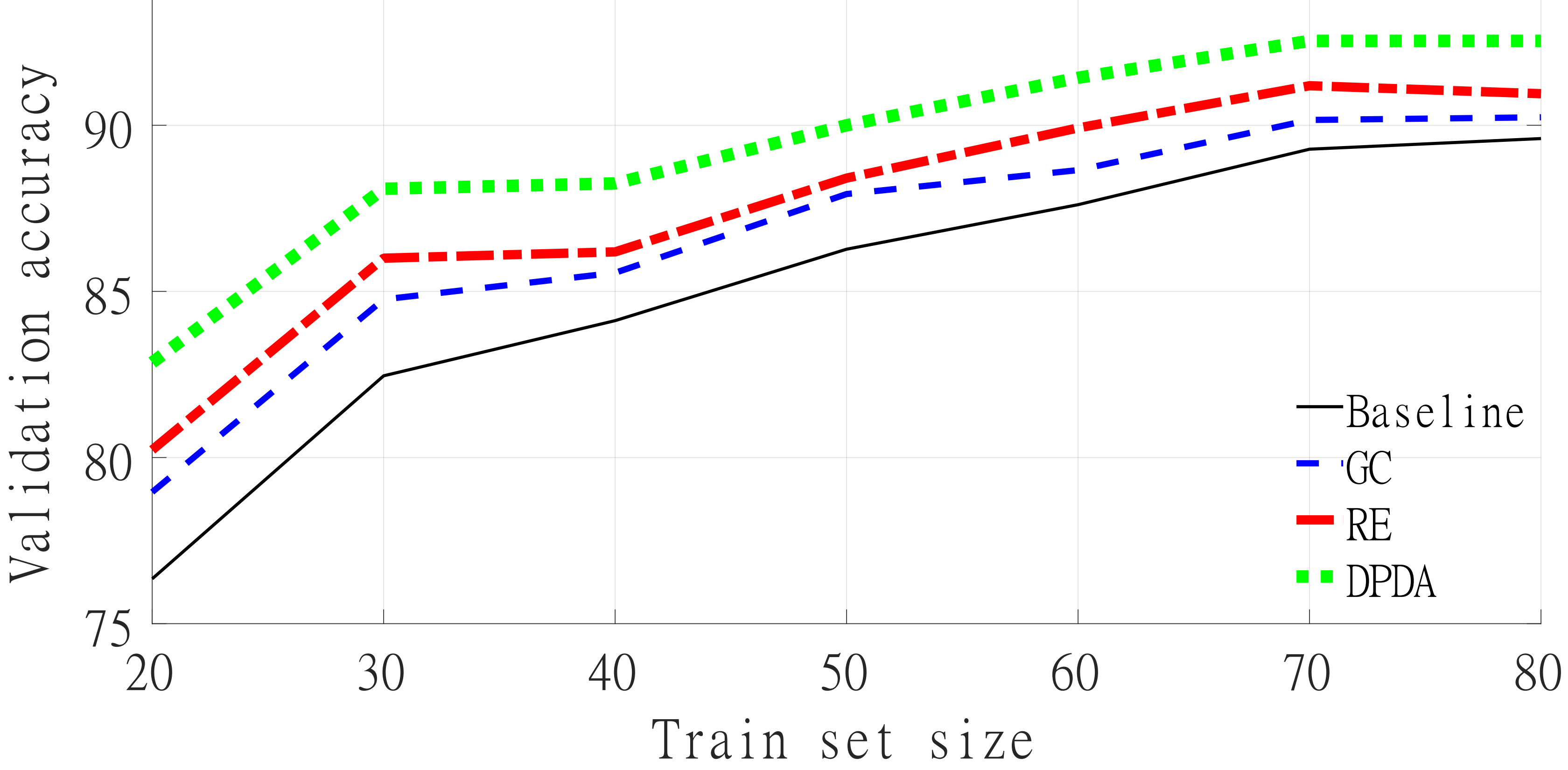

Supplement: Supplemental Information 1 [file peerj-cs-07-571-s001.pdf]

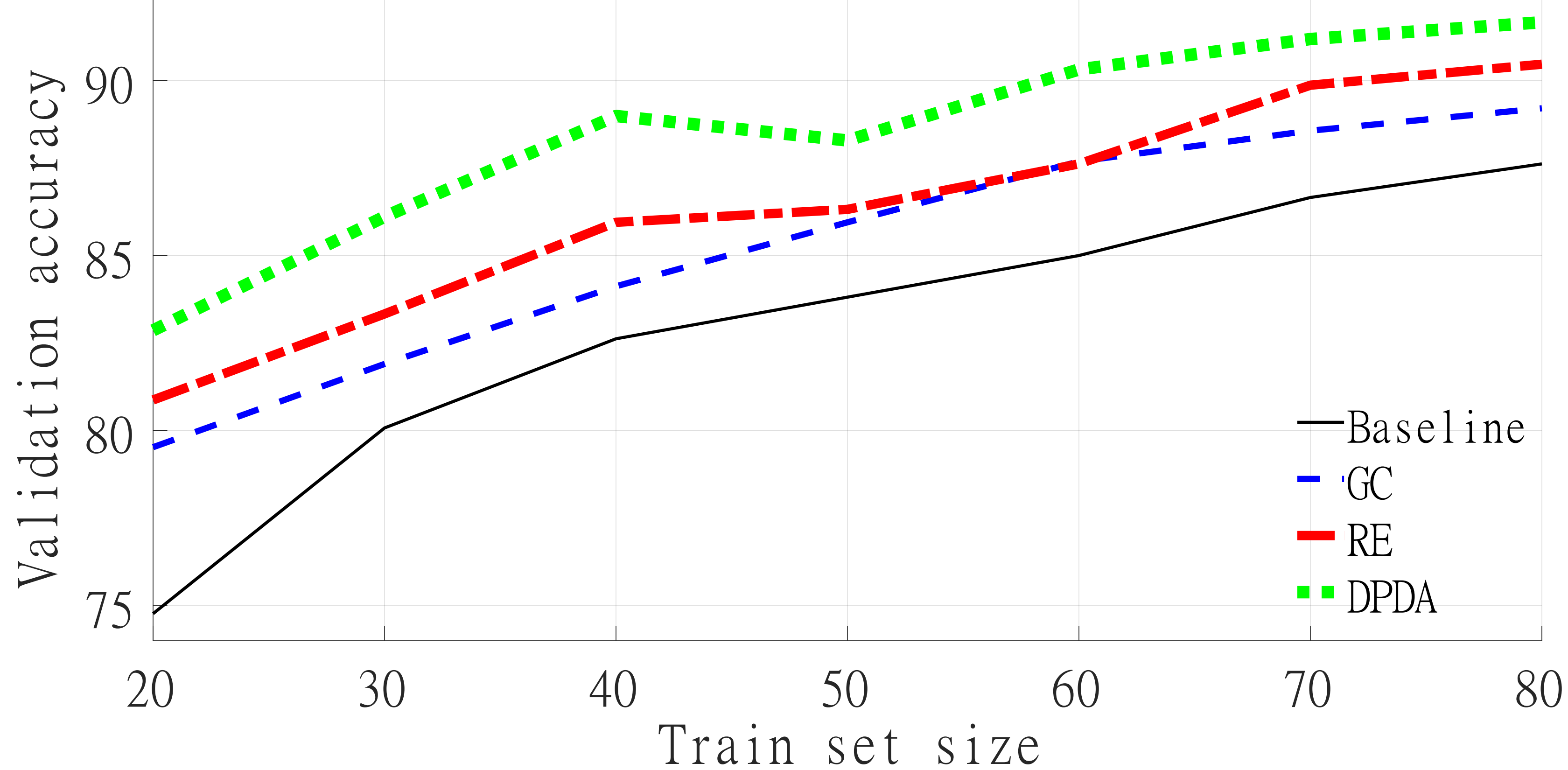

Supplement: Supplemental Information 2 [file peerj-cs-07-571-s002.pdf]

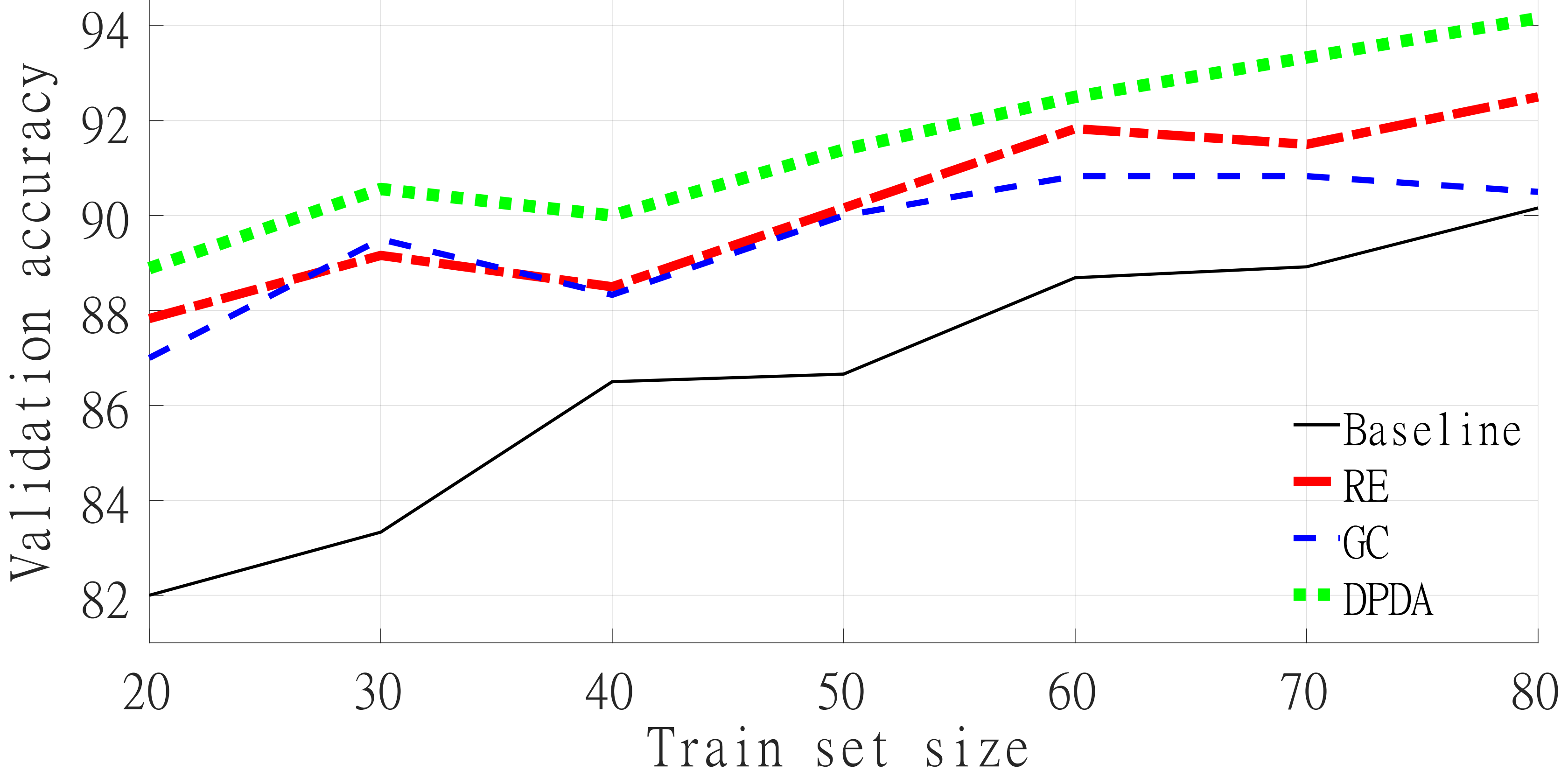

Supplement: Supplemental Information 3 [file peerj-cs-07-571-s003.pdf]

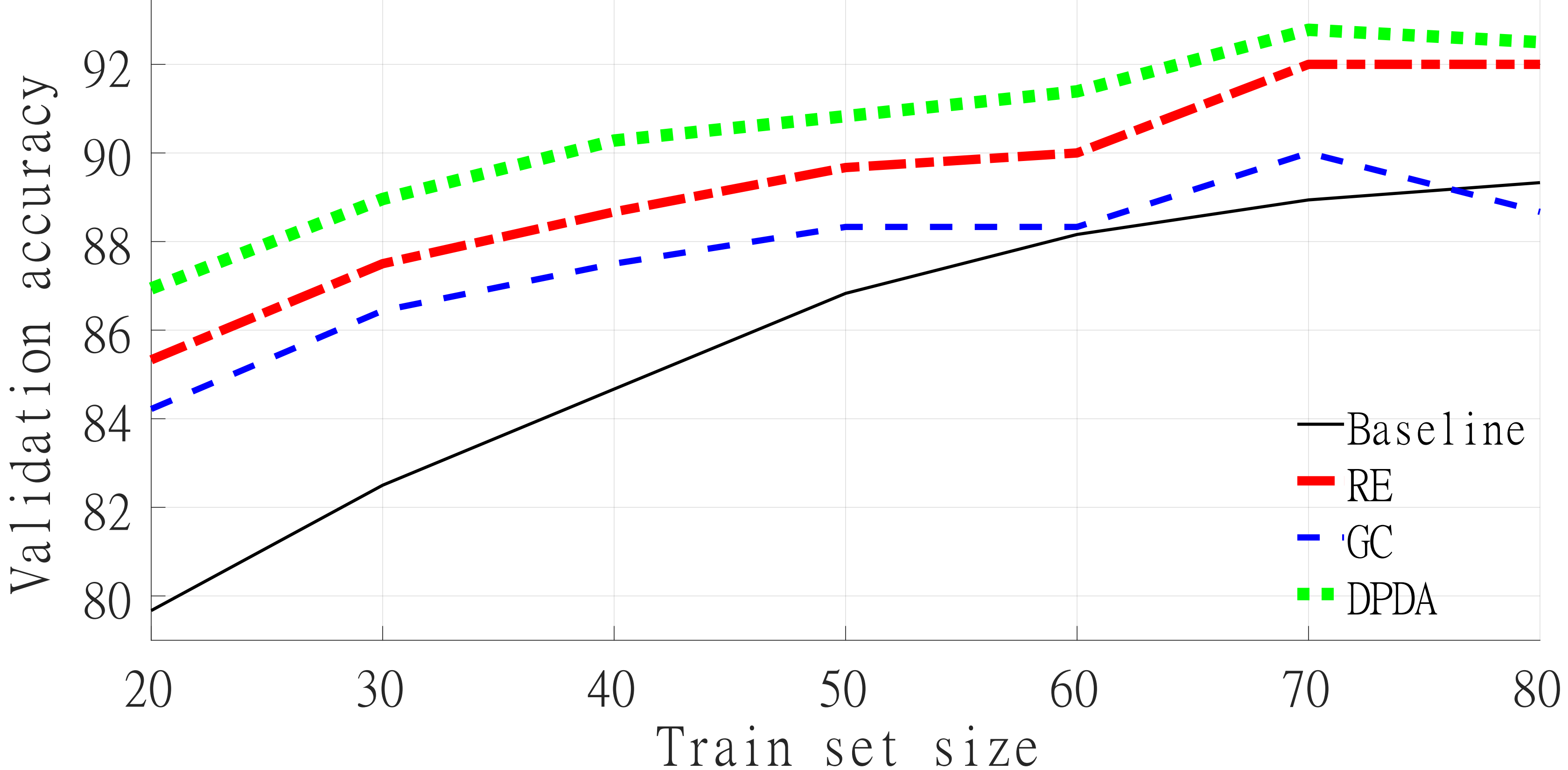

Supplement: Supplemental Information 4 [file peerj-cs-07-571-s004.pdf]

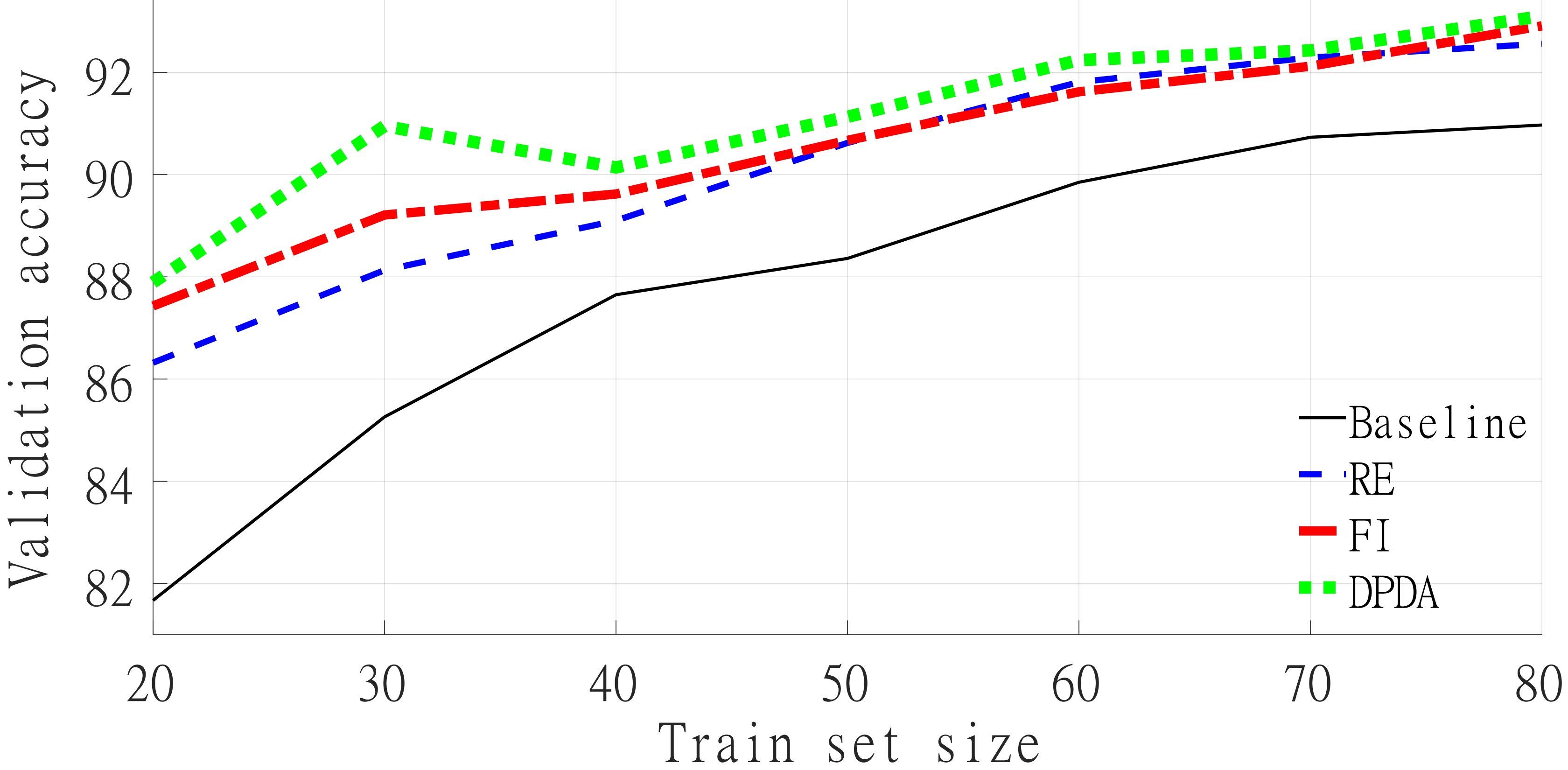

Supplement: Supplemental Information 5 [file peerj-cs-07-571-s005.pdf]

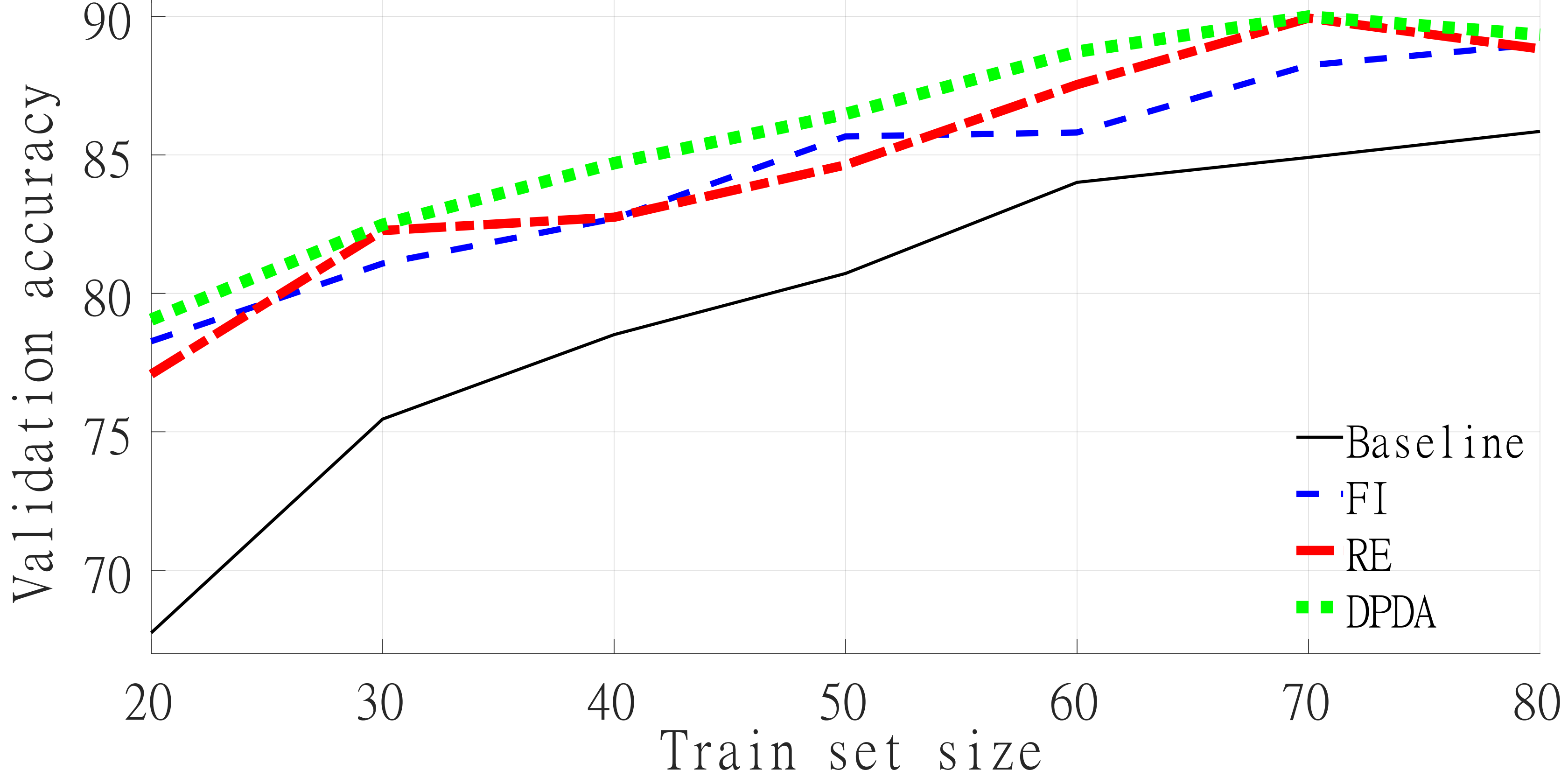

Supplement: Supplemental Information 6 [file peerj-cs-07-571-s006.pdf]
